# A novel cold-cutting direct-deployment stent for endoscopic ultrasound-guided biliary drainage: a porcine feasibility study

**DOI:** 10.1055/a-2885-8346

**Published:** 2026-07-07

**Authors:** Xuelian Gao, Yaoyuan Wang, Zhi Tang, Yue Li

**Affiliations:** 1 Endoscopy center 89346 Guangdong Provincial People’s HospitalGuangzhouGuangdongChina; 2 Research and Development Department 479749 Micro Tech Nanjing Co LtdNanjingJiangsuChina

Endoscopic ultrasound-guided biliary drainage (EUS-BD), including EUS-guided hepaticogastrostomy (EUS-HGS) and EUS-guided choledochoduodenostomy (EUS-CDS), is widely performed for malignant biliary obstruction following failed or difficult endoscopic retrograde cholangiopancreatography. 1​ Available biliary stents include conventional plastic stents, self-expandable metal stents (SEMSs), and lumen-apposing metal stents (LAMSs). However, plastic stents lack durable patency and, together with conventional SEMSs, require a cumbersome three-stage workflow (puncture, predilation, and deployment). 2​ Despite the single-step delivery of LAMSs, they are incompatible with EUS-HGS and carry inherent periprocedural risks from hot electrocautery, such as bleeding and perforation. 3​

**Fig. 1:**
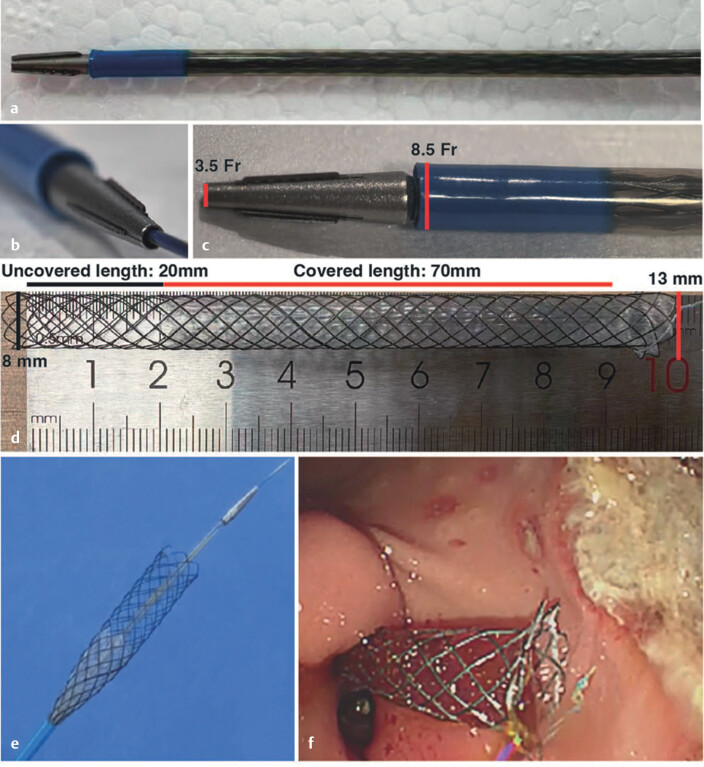
A novel cold- cutting direct-deployment biliary stent. ( **a** ) Gross appearance of the stent. ( **b** ) Distal end of the stent showing a cold-cutting tip with three blades spaced 120° circumferentially. ( **c** ) The enlarged view of the stent. ( **d** ) The partially covered self-expandable metal stent. ( **e** ) Schematic illustration of the deployment process of the stent. ( **f** ) Endoscopic image showing successful stent placement with visible bile flow.

To overcome these limitations, we developed a novel cold-cutting direct-deployment metal stent for both EUS-CDS and EUS-HGS, which streamlines the procedure ( Fig. 1a ). The integrated device comprises a cold-cutting tip with three blades spaced 120° circumferentially and a partially covered SEMS ( Fig. 1b–e ). The rationale for developing this design is to create a delivery pathway for stent implantation using mechanical cutting instead of thermal cutting. We report the first in vivo feasibility validation of this novel stent for EUS-CDS in a porcine biliary dilatation model.

The operation steps were as follows ( Video 1 ). First, under EUS guidance, we punctured the dilated common bile duct with a 19 G needle and confirmed the position with contrast. Next, we advanced and coiled a guidewire. Then, we withdrew the needle and exposed the cold cutting tip of the stent. Finally, we advanced the stent over the guidewire to cut through the duodenal and bile duct walls, deployed it by withdrawing the sheath (distal in the CBD and proximal in the duodenum), and endoscopically confirmed bile flow ( Fig. 1f ). After EUS-CDS, the pig exhibited no postoperative complications, supporting the safety of the procedure.

**Video 1** Successful EUS-guided choledochoduodenostomy (EUS-CDS) using the novel cold-cutting direct-deployment stent in a porcine biliary dilatation model.


In conclusion, this study demonstrates the technical feasibility of this cold-cutting direct-deployment metal stent for EUS-CDS, which suggests that this stent represents a promising alternative device for clinical EUS-BD.

Endoscopy_UCTN_Code_TTT_1AS_2AH
